# Treatment Selection and Survival Outcomes in Locally Advanced Proximal Gastric Cancer: A National Cancer Data Base Analysis

**DOI:** 10.3389/fonc.2020.537051

**Published:** 2020-09-25

**Authors:** Song Tang, Fangfang Liu, Yumin Li, Lulu Zhao, Xiang Wang, Sajid A. Khan, Yingtai Chen, Yawei Zhang

**Affiliations:** ^1^National Cancer Center/National Clinical Research Center for Cancer/Cancer Hospital, Chinese Academy of Medical Sciences and Peking Union Medical College, Beijing, China; ^2^Lanzhou University Second Hospital, Lanzhou, China; ^3^Fifth Medical Center of Chinese PLA General Hospital, Beijing, China; ^4^Department of Surgery, Yale School of Medicine, New Haven, CT, United States; ^5^Department of Environmental Health Sciences, Yale School of Public Health, New Haven, CT, United States

**Keywords:** national cancer data base, locally advanced proximal gastric cancer, proximal gastrectomy, total gastrectomy, long-term survival

## Abstract

**Background:** We aimed to assess long-term survival between locally advanced proximal gastric cancer (LAPGC) patients who underwent proximal gastrectomy (PG) and those who underwent total gastrectomy (TG) to evaluate the optimal extent of resection and adjuvant therapy.

**Materials and Methods:** Patients diagnosed with locally advanced proximal gastric adenocarcinoma were selected from the National Cancer Data Base (2004–2015) in America. Survival analysis was performed via Kaplan-Meier and Cox proportional hazards models.

**Results:** A total of 4,381 eligible patients were identified, 1,243 underwent PG and 3,138 underwent TG. Patients in TG group had a poor prognosis (hazard ratio [HR] = 1.13, 95% confidence interval [CI]: 1.03–1.25) compared with those in PG group. Moreover, postoperative chemoradiation therapy was associated with improved overall survival compared to surgery alone (HR = 0.71, 95% CI: 0.53–0.97) in LAPGC patients who had PG, while preoperative chemotherapy (HR = 0.74, 95% CI: 0.59–0.92) was associated with improved survival among patients who had TG.

**Conclusions:** Our study suggested that LAPGC patients underwent PG experienced better long-term outcomes than those underwent TG. It also suggested that multimodality treatment of LAPGC, including preoperative chemotherapy followed by TG or postoperative chemotherapy followed by PG, should be considered to achieve better long-term outcomes.

## Introduction

Gastric cancer (GC) is the fifth most common malignancy and the third leading cause of cancer-related mortality worldwide (1). While its overall incidence appears to be decreasing, there has been a dramatic rise in the incidence of proximal gastric cancer (PGC) (2). The shift in GC subsite has renewed interest in the management of PGC with a focus on the optimal extent of resection and adjuvant therapy.

Proximal gastrectomy (PG) and total gastrectomy (TG) are the most common surgical approaches for PGC. For early stage PGC, PG has been generally accepted by most surgeons for its comparable oncological radicality and safety with TG (3–5). The newly published “Japanese Gastric Cancer Treatment Guidelines 2018” also recommends that PG is suitable for early stage diseases (6). However, no consensus has been reached regarding which procedure should be selected for locally advanced PGC (LAPGC). Several studies investigated overall survival (OS) of LAPGC patients who underwent TG or PG and reached inconsistent results. Some studies (3, 5, 7–21) reported that TG and PG had similar OS, whereas other studies (22–25) showed that TG was associated with better 5-year OS than PG. Moreover, some studies even found that the prognosis of LAPGC patients undergoing PG was significantly better than those undergoing TG (9, 26). Deficiently, these published studies generally included limited number of patients ranging from 45 to 423. On the other hand, the optimum treatment strategy of neoadjuvant or adjuvant therapy targeted on LAPGC patients was not fully discussed in previous studies.

Here, we analyzed data from the American College of Surgeons (ACS) National Cancer Database (NCDB) to compare the OS of PGC patients who underwent PG to those who underwent TG, in order to determine whether the extent of resection for LAPGC affect prognosis and provides evidence for the development of guiding strategies for LAPGC patients.

## Materials and Methods

### Patient Population

Data were abstracted from the NCDB 2004-2015. The NCDB is a clinical oncology database sourced from hospital registry data that are collected from more than 1,500 Commission on Cancer (CoC)-accredited facilities. Eligible patients were LAPGC according to the International Classification of Diseases for Oncology codes (defined by C16.33, C16.41, C16.42, C16.52, and C16.62) and underwent definitive gastrectomy. Patients were further restricted to adenocarcinoma histology (defined by 8,140, 8,141, 8,144, 8,146, 8,147, 8,255, 8,260, 8,262, 8,310, 8,480, 8,481, 8,490, 8,510, 8,560, 8,562, and 8,570–8,576). According to the American Joint Committee on Cancer 8th (27), the locally advanced gastric cancer (LAGC) patients were defined as (1) patients treated with neoadjuvant treatment whose clinical T ≥2, *N* = any, M = 0, or whose T = any, N ≥1, M = 0, or (2) patients without neoadjuvant treatment whose pathological T ≥2, *N* = any, M = 0, or whose pathological T = any, N ≥1, M = 0. The study was exempt from the approval by the Yale Institutional Review Board as a secondary data analysis.

### Statistical Analyses

Patient demographics and clinical characteristics included age, gender, race, Hispanic ethnicity, insurance status, median income (calculated by the NCDB based on patient's zip code), facility location, facility type, distance (from patient's zip code to hospital reporting the case), year of diagnosis, Charlson Deyo score, tumor grade, clinical stage, pathological stage, number of lymph nodes examined, number of lymph nodes positive, scope of regional lymph node surgery, surgical margins, surgical inpatient stay, 30-day unplanned readmission after surgical discharge, and treatment. The sequence of chemotherapy was determined using the sequence of systemic therapy in relationship to surgery. Neoadjuvant therapy (NAT) was defined as preoperative therapies including chemotherapy and/or radiation therapy. Adjuvant therapy (AT) was defined as postoperative therapies including chemotherapy and/or radiation therapy. OS was defined as the interval between the date of diagnosis and the date of death or last contact.

Student's *t*-test for continuous variables and chi-square test for categorical variables were performed to compare patients' characteristics between PG and TG groups. Kaplan–Meier and log-rank test was used to examine OS by different treatment groups (28). Multivariate Cox regression models were employed to estimate hazard ratio (HR) and 95% confidence intervals (CI). The proportionality assumption of the cox-regression was checked by including a time-varying covariate, an interaction between the covariate and the event time. Confounding variables were selected through stepwise. Several clinically significant variables were forced into the final models although they were not statistically significant. The adjusted confounding variables included age, sex, race, Hispanic ethnicity, Charlson Deyo score, insurance status, year of diagnosis, median income, facility location, facility type, distance, tumor grade, scope of regional lymph node surgery, surgical margin, surgical inpatient stay, 30-day unplanned readmission. The adjustment for NAT and/or AT was only in the model for OS by surgical approaches. A *p* < 0.05 was considered statistically significant and all tests were two-sided. Sensitivity analysis was performed by excluding patients who died within 90 days after primary surgery in order to account for immortal time bias.

All analyses were conducted using SAS statistical software v9.3 (SAS Institute, Inc., Cary, NC).

## Results

### Cohort Characteristics

A total of 4,381 LAPGC patients with adenocarcinoma were identified, and 1,243 patients (28.37%) underwent PG and 3,138 (71.63%) underwent TG (Table 1). Approximately two thirds of the patients were aged 50–74 years at diagnosis, with a mean diagnosis age of 64.43 ± 11.57 years. Majority of the cases were male (76.69%) and white (86.56%). Compared with patients underwent TG, patients underwent PG were more likely to be older, male, and white. Patients in PG group were more likely to have a higher proportion of R0 resection (88.33 vs. 84.00%, *p* < 0.01) and well or moderately differentiated tumor grade, whereas patients in TG group were more likely to have a higher proportion of more than 15 nodes examined (53.98 vs. 35.32%, *p* < 0.01) and nodes positive (67.30 vs. 63.07%, *p* < 0.01). Carlson scores, surgical inpatient stay, and 30-day unplanned readmission were not significantly different between the two groups (*p* > 0.05).

**Table 1:** Patient characteristics.

**Characteristic**	**Total (*****n*** **=** **4,381)**		**PG (*****n*** **=** **1,243)**		**TG (*****n*** **=** **3,138)**		P^*^value
	**Number**	**%**	**Number**	**%**	**Number**	**%**	
**Age (y)**							
<50	445	10.16	101	8.13	344	10.96	
50–64	1,610	36.75	439	35.32	1,171	37.32	
65–74	1,434	32.73	436	35.08	998	31.80	
≥75	892	20.36	267	21.48	625	19.92	0.0074
**Gender**							
Male	3,360	76.69	993	79.89	2,367	75.43	
Female	1,021	23.31	250	20.11	771	24.57	0.0017
**Race**							
White	3,792	86.56	1,150	92.52	2,642	84.19	
Black	297	6.78	47	3.78	250	7.97	
Other	292	6.67	46	3.70	246	7.84	<0.0001
**Hispanic ethnicity**							
Non-hispanic	3,882	88.61	1,123	90.35	2,759	87.92	
Hispanic	285	6.51	70	5.63	215	6.85	
Unknown	214	4.88	50	4.02	164	5.23	0.1154
**Insurance**							
Uninsured	96	2.19	22	1.77	74	2.36	
Private Insurance	1,871	42.71	536	43.12	1,335	42.54	
Medicaid	224	5.11	52	4.18	172	5.48	
Medicare	2,082	47.52	617	49.64	1,465	46.69	
Other	51	1.16	11	0.88	40	1.27	
Unknown	57	1.30	5	0.40	52	1.66	
**Median income ($)**							
<38,000	710	16.21	188	15.12	522	16.63	
38,000–47,999	975	22.26	293	23.57	682	21.73	
48,000–62,999	1,152	26.30	314	25.26	838	26.70	
≥63,000	1,469	33.53	431	34.67	1,038	33.08	
Unknown	75	1.71	17	1.37	58	1.85	0.2661
**Circle distance (miles)**							
<50	3,480	79.43	1,009	81.17	2,471	78.74	
>50	829	18.92	217	17.46	612	19.50	
Unknown	72	1.64	17	1.37	55	1.75	0.1061
**Facility location**							
Northeast	1,100	25.11	284	22.85	816	26.00	
Midwest	1,100	25.11	345	27.76	755	24.06	
South	1,395	31.84	373	30.01	1,022	32.57	
West	691	15.77	227	18.26	464	14.79	
Other	95	2.17	14	1.13	81	2.58	0.0008
**Facility type**							
Non-academic	2,146	48.98	670	53.90	1,476	47.04	
Academic	2,140	48.85	559	44.97	1,581	50.38	
Unknown	95	2.17	14	1.13	81	2.58	0.0002
**Tumor location**							
Cardia	3,732	85.19	1,130	90.91	2,602	82.92	
Fundus	649	14.81	113	9.09	536	17.08	<0.0001
**Charlson score**							
0	3,000	68.48	836	67.26	2,164	68.96	
1	1,047	23.90	310	24.94	737	23.49	
2	253	5.77	73	5.87	180	5.74	
3	81	1.85	24	1.93	57	1.82	0.7395
**Clinical T**							
cT0	12	0.27	4	0.32	8	0.25	
cT1	247	5.64	92	7.40	155	4.94	
cT2	689	15.73	222	17.86	467	14.88	
cT3	1,854	42.32	506	40.71	1,348	42.96	
cT4	165	3.77	31	2.49	134	4.27	
cTx	1,414	32.28	388	31.21	1,026	32.70	0.0001
**Clinical N**							
cN0	1,471	33.58	456	36.69	1,015	32.35	
cN1	1,396	31.86	390	31.38	1,006	32.06	
cN2	323	7.37	78	6.28	245	7.81	
cN3	87	1.99	16	1.29	71	2.26	
cNx	1,104	25.20	303	24.38	801	25.53	0.0074
**Clinical M**							
cM0	4,381	100.00	1243	100.00	3,138	100.00	
cM1	0	0.00	0	0.00	0	0.00	~
**CTNM**							
I	554	12.65	197	15.85	357	11.38	
II	1,029	23.49	290	23.33	739	23.55	
III	1,298	29.63	344	27.67	954	30.40	
IV	19	0.43	4	0.32	15	0.48	
Unknown	1,481	33.81	408	32.82	1,073	34.19	0.0017
**Pathologic T**							
pT0	246	5.62	79	6.36	167	5.32	
pT1	350	7.99	116	9.33	234	7.46	
pT2	1,206	27.53	386	31.05	820	26.13	
pT3	1,917	43.76	506	40.71	1,411	44.96	
pT4	324	7.40	41	3.30	283	9.02	
pTx	338	7.72	115	9.25	223	7.11	<0.0001
**Pathologic N**							
pN0	1,470	33.55	442	35.56	1,028	32.76	
pN1	1,430	32.64	441	35.48	989	31.52	
pN2	702	16.02	166	13.35	536	17.08	
pN3	441	10.07	73	5.87	368	11.73	
pNx	338	7.72	121	9.73	217	6.92	<0.0001
**Pathologic M**							
pM0	4,381	100.00	1,243	100.00	3,138	100.00	
pM1	0	0.00	0	0.00	0	0.00	~
**PTNM**							
I	953	21.75	324	26.07	629	20.04	
II	1,530	34.92	431	34.67	1,099	35.02	
III	1,292	29.49	287	23.09	1,005	32.03	
IV	0	0.00	0	0.00	0	0.00	
Unknown	606	13.83	201	16.17	405	12.91	<0.0001
**Number of nodes examined**							
0–15 nodes	2,132	48.66	741	59.61	1,391	44.33	
>15 nodes	2,133	48.69	439	35.32	1,694	53.98	
Unknown	76	1.73	23	1.85	53	1.69	<0.0001
**Number of nodes positive**							
0 nodes	1,485	33.90	459	36.93	1,026	32.70	
1–2 nodes	1,061	24.22	330	26.55	731	23.30	
3–6 nodes	864	19.72	231	18.58	633	20.17	
7–15 nodes	623	14.22	140	11.26	483	15.39	
16 or more nodes	200	4.57	26	2.09	174	5.54	
Unknown	148	3.38	57	4.59	91	2.90	<0.0001
**Scope of regional lymph node surgery**							
No	78	1.78	38	3.06	40	1.27	
Yes	4,284	97.79	1,200	96.54	3,084	98.28	
Unknown	19	0.43	5	0.40	14	0.45	<0.0001
**Tumor grade**							
Well	168	3.83	55	4.42	113	3.60	
Moderately	1,316	30.04	414	33.31	902	28.74	
Poorly	2,512	57.34	663	53.34	1,849	58.92	
Undifferentiated; anaplastic	103	2.35	22	1.77	81	2.58	
Unknown	282	6.44	89	7.16	193	6.15	0.0018
**Surgical margin**							
R0	3,734	85.23	1,098	88.33	2,636	84.00	
R1	355	8.10	89	7.16	266	8.48	
R2	217	4.95	35	2.82	182	5.80	
Unknown	75	1.71	21	1.69	54	1.72	<0.0001
**Surgical inpatient stay (days)**							
0–5	423	9.66	117	9.41	306	9.75	
6–7	562	12.83	156	12.55	406	12.94	
8–11	1,488	33.96	421	33.87	1,067	34.00	
≥12	1,468	33.51	413	33.23	1,055	33.62	
Unknown	440	10.04	136	10.94	304	9.69	0.9917
**30-day unplanned readmission**							
No unplanned readmission	3,979	90.82	1,124	90.43	2,855	90.98	
Unplanned readmission	252	5.75	72	5.79	180	5.74	
Unknown	150	3.42	47	3.78	103	3.28	0.9120
**Age at diagnosis (y)**							
Mean	64.43		65.42		64.21		
SD	11.57		10.91		11.66		0.0016

*PG, proximal gastrectomy; TG, total gastrectomy; SD, standard deviation*.

* *Comparisons between PG and TG group*.

### Survival Outcomes for Patients Who Underwent PG and TG

**Figure 1:**
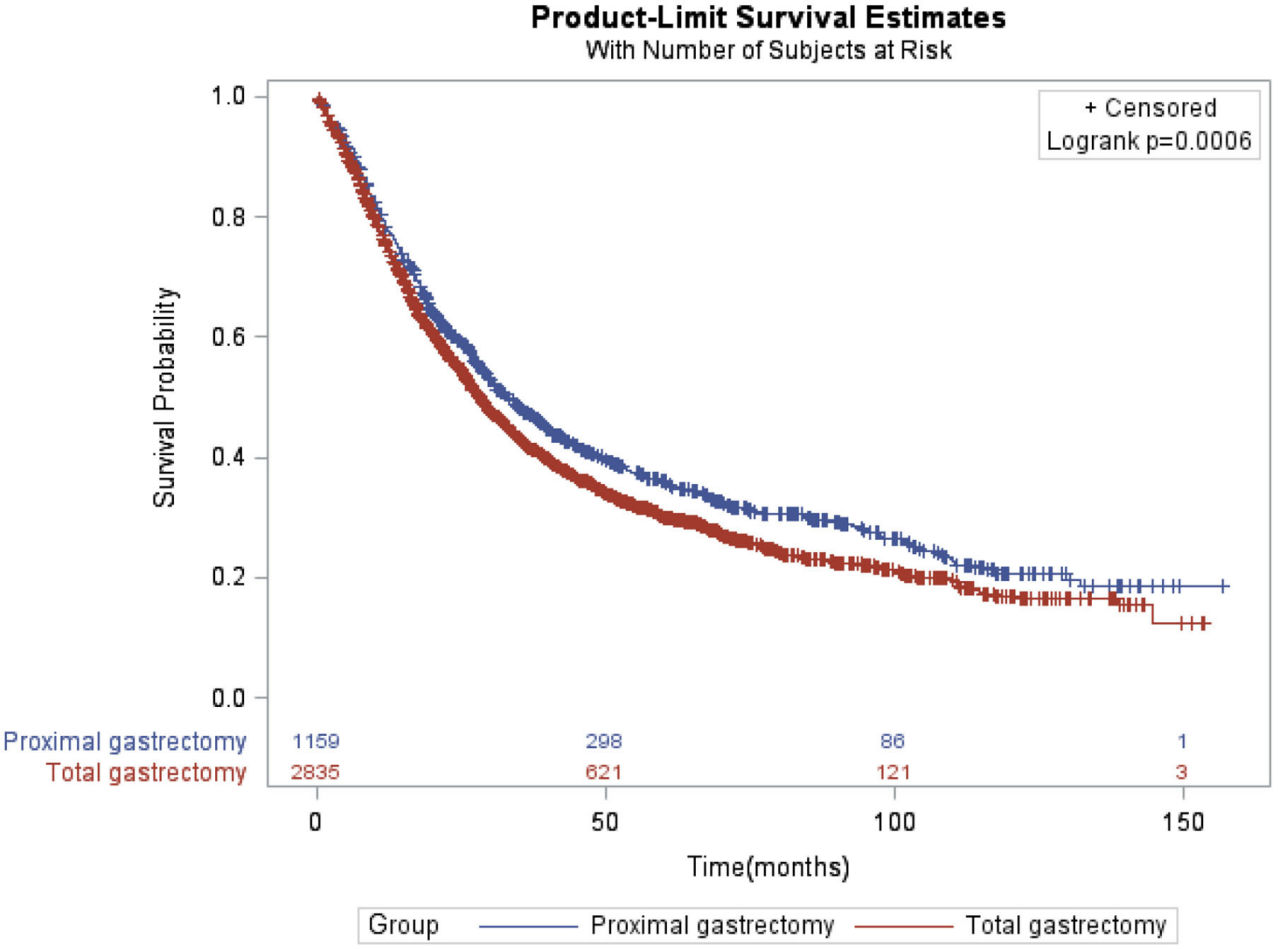
Kaplan-Meier of overall survival of locally advanced proximal gastric cancer patients by proximal and total gastrectomy.

Compared with patients in TG group, patients in PG group had longer OS as shown in Figure 1 (*p* = 0.0006). The median survival time was 32.99 months (95% CI: 30.06–37.03 months) for PG group and 28.19 months (95% CI: 26.64–29.57 months) for TG group. The 3- and 5-year survival rates were 47.40 and 35.94% for PG and 42.06 and 30.14% for TG, respectively). After controlling for confounding variables, patients who underwent TG had poor OS compared to patients who underwent PG (HR = 1.13, 95% CI: 1.03–1.25; *p* = 0.0109) (Table 2).

**Table 2:** Multivariate analysis of locally advanced proximal gastric cancer patients by surgical approach.

			**Adjusted**			
			**HR**	**95%CI**		***P*-value**
**Prognostic Factors**	**Number**	**%**		**Lower**	**Upper**	
PG	1,025	29.45	ref			
TG	2,456	70.55	1.133	1.029	1.246	0.0109

*Adjusted for age, gender, race, Hispanic ethnicity, Charlson Deyo score, insurance status, year of diagnosis, median income, facility location, facility type, distance, tumor grade, scope of regional lymph node surgery, surgical margin, surgical inpatient stay, 30-day unplanned readmission, and neoadjuvant and/or adjuvant therapy*.

*HR, hazard ratio; CI, confidence intervals*.

### Survival Outcomes for Patients With Different Adjuvant Therapies in Locally Advanced Stage

**Figure 2:**
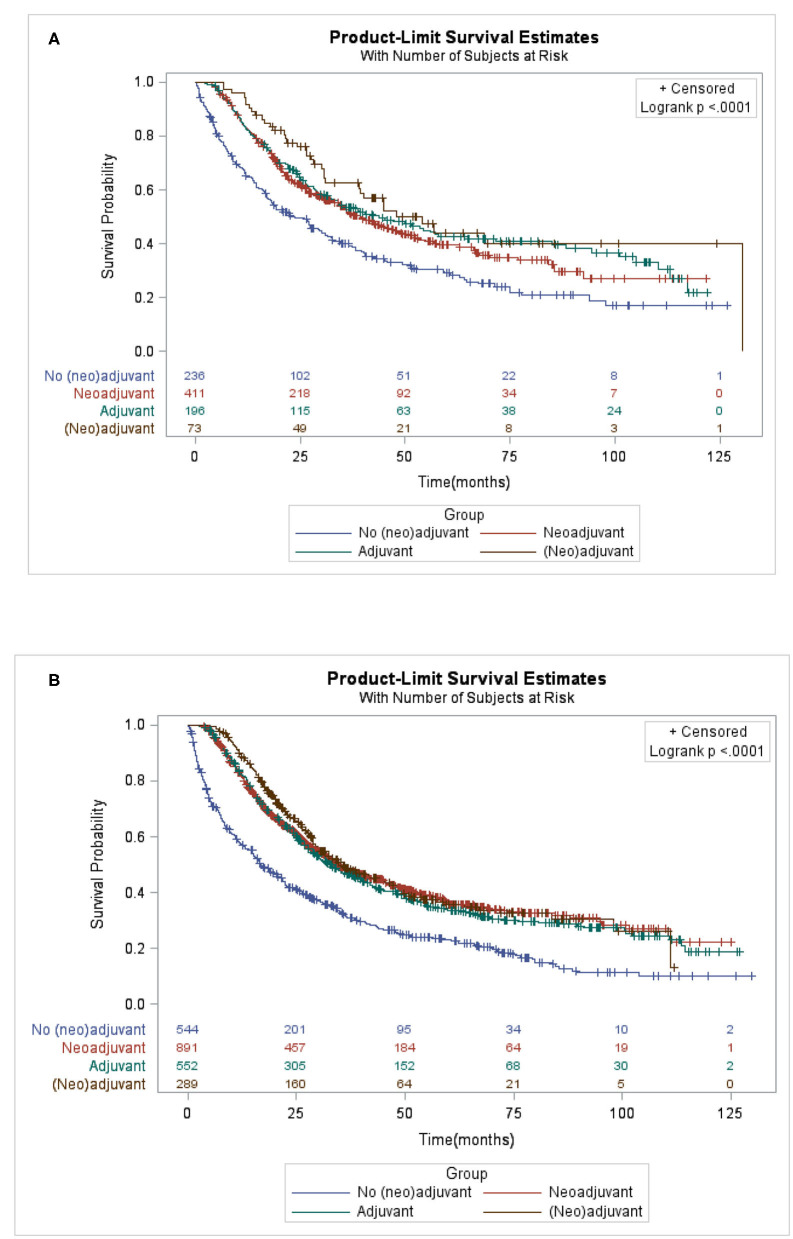
Kaplan-Meier of overall survival of locally advanced proximal gastric cancer patients who underwent **(A)** proximal gastrectomy **(B)** or total gastrectomy by different adjuvant therapy.

Patients who received AT, NAT only, or NAT plus AT had improved OS compared with patients who underwent gastrectomy alone regardless of PG (Figure 2A, *p* < 0.0001) or TG (Figure 2B, *p* < 0.0001). The median survival time for patients who underwent PG were 23.66, 39.49, 43.83, and 54.08 months in gastrectomy alone, NAT, AT, and NAT plus AT, respectively. The corresponding median survival time for patients who underwent TG were 16.82, 34.69, 32.95, and 35.81 months, respectively. No significant differences in survival benefits between different adjuvant therapies.

After controlling for confounding variables (Table 3), only AT was associated with improved OS compared to surgery alone in PG group (HR = 0.70, 95% CI: 0.52–0.92; *p* = 0.0114). However, there was no significant survival benefit for various adjuvant therapies among patients who underwent TG.

**Table 3:** Multivariate analysis for locally advanced proximal gastric cancer patients by neoadjuvant and adjuvant therapy.

	**PG**						**TG**					
			**Adjusted**						**Adjusted**			
			**HR**	**95%CI**		***P*-value**			**HR**	**95%CI**		***P*-value**
**Prognostic factors**	**Number**	**%**		**Lower**	**Upper**		**Number**	**%**		**Lower**	**Upper**	
**THERAPY**												
No NAT/AT	187	22.86	ref				401	20.04	ref			
NAT	372	45.48	0.813	0.624	1.059	0.1244	790	39.48	0.859	0.726	1.017	0.0782
AT	187	22.86	0.695	0.524	0.921	0.0114	530	26.49	0.959	0.809	1.137	0.6296
NAT plus AT	72	8.80	0.7	0.466	1.052	0.0863	280	13.99	0.93	0.750	1.153	0.5065

*Adjusted for age, gender, race, Hispanic ethnicity, CharlsonDeyo score, insurance status, year of diagnosis, median income, facility location, facility type, distance, tumor grade, scope of regional lymph node surgery, surgical margin, surgical inpatient stay, 30-day unplanned readmission. NAT, neoadjuvant therapy; AT, adjuvant therapy; HR, hazard ratio; CI, confidence intervals*.

We further analyzed the data by detailed therapies (Table 4), among patients who underwent PG, postoperative CT plus RT (HR = 0.71, 95% CI: 0.53–0.97; *p* = 0.0316) was associated with improved survival compared to surgery alone. Among patients who underwent TG, only preoperative CT (HR = 0.74, 95% CI: 0.59–0.92; *p* = 0.0078) was associated with improved survival compared to surgery alone.

**Table 4:** Multivariate analysis for locally advanced proximal gastric cancer patients with detailed neoadjuvant and adjuvant therapy.

	**PG**						**TG**					
			**Adjusted**						**Adjusted**			
			**HR**	**95%CI**		***P*-value**			**HR**	**95%CI**		***P*-value**
**Prognostic factors**	**Number**	**%**		**Lower**	**Upper**		**Number**	**%**		**Lower**	**Upper**	
No adjuvant	187	22.92	ref				398	20.01	ref			
Pre-op chemo only	42	5.15	0.99	0.614	1.598	0.9680	257	12.92	0.738	0.590	0.922	0.0076
Post-op chemo only	49	6.00	0.631	0.398	1.000	0.0502	169	8.50	0.938	0.740	1.188	0.5943
pre and post chemo only	19	2.33	0.468	0.202	1.084	0.0763	127	6.39	0.807	0.602	1.082	0.1522
Pre-op chemo and rad	326	39.95	0.785	0.598	1.029	0.0798	528	26.55	0.916	0.762	1.101	0.3496
Pre-op rad and chemo and Post-op chemo	34	4.17	0.880	0.528	1.466	0.6235	64	3.22	1.036	0.725	1.480	0.8455
Pre-op chemo and Post-op rad	7	0.86	0.995	0.357	2.773	0.9920	41	2.06	1.011	0.653	1.565	0.9612
Post-op chemo and rad	133	16.30	0.714	0.526	0.971	0.0316	350	17.60	0.949	0.785	1.146	0.5846
Pre-op chemo and Post-op rad and chemo	8	0.98	0.501	0.153	1.636	0.2521	34	1.71	0.868	0.537	1.401	0.5615
Others	11	1.35	0.702	0.303	1.628	0.4102	21	1.06	1.677	1.047	2.684	0.0313

*Adjusted for age, gender, race, Hispanic ethnicity, Charlson Deyo score, insurance status, year of diagnosis, median income, facility location, facility type, distance, tumor grade, scope of regional lymph node surgery, surgical margin, surgical inpatient stay, 30-day unplanned readmission*.

*CT, chemotherapy; RT, radiotherapy; Pre-op, preoperative; Post-op, postoperative; HR, hazard ratio; CI, confidence intervals*.

*Others included Pre-op RT only, Pre-op RT plus Post-op CT, and Post-op RT only*.

## Discussion

Our study suggested an improved survival benefit of PG compared to TG among patients diagnosed with LAPGC. In contrast to early studies that have reported no differences in survival outcomes between the two surgical procedures (3, 5, 7–21) or a better survival was associated with TG procedure (22–25). Varying patients' characteristics in different study populations might account for the inconsistent results. A meta-analysis (22) reported no difference in survival between TG and PG groups among LAPGC patients, which was inconsistent with our study results. A possible explanation was that patients in TG group had a higher proportion (58.92%) of poorer tumor grade in our cohort whereas the patients in early studies (9–12, 14) had a lower proportion (ranging from 27.5 to 52%) of poorer tumor grade. Another possible explanation was that confounding factors, such as adjuvant or neoadjuvant therapies were not controlled in early studies.

Neoadjuvant therapy or adjuvant therapy have been developed over the last decades as part of a multimodality treatment in order to improve survival for LAPGC patients with gastrectomy (29). However, no consensus has been reached regarding the optimal choice. Our study revealed a better prognosis for PGC patients given AT compared to gastrectomy alone only in PG group, but no significant survival benefits of AT in TG group. Previous studies have reported improved patient survival with the addition of AT or NAT compared to surgery alone (30, 31). Surgery does not always result in complete resection, which is likely to cause recurrence and metastasis and influence the long-term outcomes (22). NAT is expected to improve the resection rate and long-term follow-up results by reducing the size of the primary lesion and controlling lymph node metastasis and micrometastasis (32). Therefor NAT has been recommended for PGC patients with advanced clinical stage. AT controls residual tumor cells following curative resection, and therefore improve the long-term follow-up results (31).

The increased use of preoperative CT for patients with PGC was a dominant trend among patients with locally advanced disease (33). Preoperative CT has several advantages, including a greater likelihood of completing treatment, a rapid improvement in tumor-related symptoms, the potential to downstage tumors, and the ability to assess response to preoperative therapy (33). In our study, survival benefit of preoperative CT only was shown for LAPGC patients who underwent TG but not those who underwent PG. The reasons for this are currently unclear and warrant further investigation. Postoperative CT or RT is delivered with an intention to reduce recurrence by controlling residual tumor cells following curative resection. Recent advances in postoperative CT have achieved considerable tumor regression in many cases of gastric cancer (6). In our study, we also observed postoperative CT with or without RT showed OS benefit only for LAPGC patients who underwent PG.

Study limitations should be considered when interpreting the study results. Although ~70% of all newly diagnosed cases of cancer in the United States were reported to the NCDB, the NCDB collects data only from Commission on Cancer–accredited hospitals which might affect the generalizability of the study results. Some treatment-related information was unavailable, including recurrence time, metastasis time, treatment intent, specific chemotherapy regimens, completion of prescribed treatment schedules, and toxicities of the received therapies. Information concerning more granular endpoints including disease specific survival, recurrence, and postoperative complications were also not available. In addition, the numbers of patients in certain specific neoadjuvant and adjuvant therapy groups were too small, which provided limited power to evaluate their effects on OS. Despite these limitations, the NCDB provides a large sample size, making this study one of the largest studies to assess long-term survival between LAPGC patients who underwent PG and TG.

In conclusion, long-term outcome disparities exist between LAPGC patients who underwent TG and those who underwent PG in the United States. Patients treated with PG had better OS than those who underwent TG, suggesting PG might be an optimal extent of resection for LAPGC patients. The study also suggested that LAPGC should be treated with multimodality treatment approach, including preoperative CT followed by TG or postoperative CT followed by PG. The findings in our study need to be verified in randomized controlled clinical trials.

## Data Availability Statement

The raw data supporting the conclusions of this article will be made available by the authors, without undue reservation, to any qualified researcher.

## Author Contributions

YL: conceptualization, methodology, and writing–review and editing. LZ: conceptualization, methodology, and writing–review. XW: writing–review and editing. SK: conceptualization, investigation, resources, and writing–review and editing. YC: conceptualization, methodology, funding acquisition, and writing–review and editing. YZ: conceptualization, methodology, investigation, resources, supervision, and writing–review and editing. ST, FL, and ST: conceptualization, methodology, and writing first draft. FL: conceptualization, methodology, data analysis, and writing–review and editing. All authors: contributed to the article and approved the submitted version.

## Conflict of Interest

The authors declare that the research was conducted in the absence of any commercial or financial relationships that could be construed as a potential conflict of interest.
